# Identification and Analysis of Axolotl Homologs for Proteins Implicated in Human Neurodegenerative Proteinopathies

**DOI:** 10.3390/genes15030310

**Published:** 2024-02-28

**Authors:** Lucas M. James, Zachary Strickland, Noah Lopez, Jessica L. Whited, Malcolm Maden, Jada Lewis

**Affiliations:** 1 Department of Neuroscience, University of Florida, Gainesville, FL 32610, USA; lucas.james@med.unc.edu (L.M.J.); z.strickland@ufl.edu (Z.S.); 2 Center for Translational Research in Neurodegenerative Disease, University of Florida, Gainesville, FL 32610, USA; 3 Evelyn F. and William L. McKnight Brain Institute, University of Florida, Gainesville, FL 32610, USA; 4 Department of Stem Cell and Regenerative Biology, Harvard University, Cambridge, MA 02138, USA; 5 The Harvard Stem Cell Institute, Cambridge, MA 02138, USA; 6 The Broad Institute of MIT and Harvard, Cambridge, MA 02142, USA; 7 Department of Biology and UF Genetics Institute, University of Florida, Gainesville, FL 32611, USA

**Keywords:** neurodegeneration, proteinopathy, axolotl, regeneration, proteins

## Abstract

Neurodegenerative proteinopathies such as Alzheimer’s Disease are characterized by abnormal protein aggregation and neurodegeneration. Neuroresilience or regenerative strategies to prevent neurodegeneration, preserve function, or restore lost neurons may have the potential to combat human proteinopathies; however, the adult human brain possesses a limited capacity to replace lost neurons. In contrast, axolotls (*Ambystoma mexicanum*) show robust brain regeneration. To determine whether axolotls may help identify potential neuroresilience or regenerative strategies in humans, we first interrogated whether axolotls express putative proteins homologous to human proteins associated with neurodegenerative diseases. We compared the homology between human and axolotl proteins implicated in human proteinopathies and found that axolotls encode proteins highly similar to human microtubule-binding protein tau (tau), amyloid precursor protein (APP), and β-secretase 1 (BACE1), which are critically involved in human proteinopathies like Alzheimer’s Disease. We then tested monoclonal Tau and BACE1 antibodies previously used in human and rodent neurodegenerative disease studies using immunohistochemistry and western blotting to validate the homology for these proteins. These studies suggest that axolotls may prove useful in studying the role of these proteins in disease within the context of neuroresilience and repair.

## 1. Introduction

Frontotemporal dementia (FTD) and Alzheimer’s disease (AD) affect over 55 million individuals worldwide and belong to a family of neurodegenerative diseases known as proteinopathies [1]. These proteinopathies are defined using neurodegeneration and pathological aggregation of one or more proteins. The aggregation of amyloid-β (Aβ) peptide into amyloid plaques is a key feature of AD [2]. Additionally, AD and some cases of FTD pathologically aggregate the microtubule-associated protein tau (tau) [3]. Post-mitotic human neurons cannot dilute harmful protein aggregates, rendering neurons especially susceptible to neurotoxicity and increasing the susceptibility of older brains to proteinopathies [4]. Although recent breakthroughs targeting amyloid-β are promising, treatments aimed at clearing these pathological aggregates have historically found little success in ameliorating neurological symptoms and combatting neurodegeneration [5,6]. Identifying neuroresilience and repair pathways and developing therapeutic strategies that exploit these pathways might transform the therapeutic landscape for neurodegenerative proteinopathies. Current approaches directed toward identifying neuroresilience and repair pathways in the CNS primarily focus on mammalian systems; however, looking beyond mammals may offer novel insights into these processes [7].

While much of the human body is considered non-regenerative, humans exhibit some regenerative capacity, such as the regeneration of uninjured skin or the endometrial lining [8,9]. Angelotti et al. define regeneration as “the ability to recreate lost or damaged tissues and organs, reestablishing their natural morphology and function” [10]. Although human nervous tissues possess stem and progenitor cells that contribute to cell turnover and tissue homeostasis, neurons are not typically replaced in response to injury or degeneration in aged humans [11]. Human neurogenesis occurs primarily during development and at very limited levels throughout adulthood in the subventricular zone and the dentate gyrus within the hippocampus [12].

In contrast, axolotls (*A. mexicanum*) possess the remarkable ability to regenerate complex biological structures in response to injuries such as appendage loss, spinal cord injury, and forebrain lesion, making this species of widespread utility and interest to many fields [13]. However, the origins of these abilities are not completely understood. The axolotl brain regenerates extensively after the removal of large quantities of tissue, specifically in the telencephalon and pallium [13,14,15]. Neurogenesis, a key element of the regenerative response, occurs at variable rates by region and intensifies in otherwise quiescent areas post-injury, such as the midbrain during dopaminergic neuron regeneration in the red-spotted newt (*Notophthalmus viridescens*), another salamander species, and the rostral telencephalon following pallial lesion in axolotls [14,16]. During regeneration of the axolotl forebrain, neural progenitor cells within the ventricular zone proliferate, and their progeny migrate throughout the telencephalon before differentiating into neurons [15]. In contrast, pallial lesions are repaired via the proliferation of activated ependymoglia and subsequent differentiation into neurons 4–11 weeks after injury [14]. While regenerated axolotl neurons are electrophysiologically healthy and functionally diverse, long-distance axonal tracts present before the injury are not fully reestablished, and it remains unclear what physiological consequences this causes to the organism [14]. The signaling factor(s) potentially involved in injury-mediated neurogenesis have not been identified nor rigorously experimentally tested in axolotls. However, in the red-spotted newt, the Sonic hedgehog signaling pathway has been implicated in the process of ependymal cells acting as neuronal progenitors in adults [16].

Interestingly, the axolotl has never been reported to develop neurodegenerative pathology upon necropsy. We initially hypothesized that axolotls might not express the same proteins that form toxic aggregates in human neurodegenerative conditions like Alzheimer’s Disease (AD). Here, we sought to determine if axolotls expressed the encoded proteins associated with human neurodegenerative diseases in determining if axolotls have the potential utility to study human proteinopathies and neuroresilience strategies that may be present to combat these diseases. The 32-billion base pair mega-genome of the axolotl was only recently sequenced [17]. We are not aware of any existing reports examining the extent to which proteins underlying AD and related disorders might be conserved in sequence, structure, expression, and, ultimately, function in the axolotl. To begin to address these issues, we identified putative axolotl sequences in Axolotl-Omics.org homologous to human *MAPT*, *APP*, *BACE1*, and their encoded proteins, which are linked to AD and/or FTD. We analyzed them using sequence alignment for conservation within protein functional domains. To determine if the encoded proteins were expressed in axolotl brain tissues, we selected well-characterized antibodies targeting epitopes that we identified as likely conserved between the human and the predicted axolotl tau and BACE1 proteins and performed immunoblotting and immunohistochemistry. Our data demonstrate for the first time that key protein epitopes in tau and BACE1 are sufficiently similar between humans and axolotls to enable the analysis of these putative proteins using currently available tools. These data support the potential viability of axolotls as a model in which the field can analyze neuroresilience and neuroregenerative mechanisms that may protect axolotls from proteinopathies and potentially yield insight into novel therapeutic strategies for these diseases.

## 2. Materials and Methods

### 2.1. Animals

Axolotls (white genotype) were obtained from the Harvard Axolotl Facility, where they were bred and raised to adulthood in separate enclosures. C57BL/6J mice were obtained from Jackson Labs and housed up to 5 animals per cage with *ad libitum* food and water on a 14-h light and 10-h dark cycle. All experiments involving animals were approved by the Institutional Animal Care and Use Committee of Harvard University (axolotls) or the University of Florida (mice) and conducted in accordance with institutional and federal regulations.

### 2.2. Identification of Putative Homologous Proteins in Axolotls and Humans

Sequences for human proteins were retrieved from NCBI RefSeq (Supplementary Table S1). Amino acid sequences were uploaded to the Axolotl-Omics.org [18] database and analyzed using the BLAST tblastn function to identify homologous axolotl-translated nucleotide sequences. These tools were created by Nowoshilow et al. to analyze the complete genome of the axolotl [17]. Using these databases, open reading frames and protein sequences of interest were identified, and accession information for these putative sequences was reported (Supplementary Table S2).

### 2.3. Putative Protein Alignment

Identified axolotl protein sequences were aligned against the corresponding human full-length proteins using the NIH BLAST blastp suite, and the results are reported in Table 1 [19,20]. Axolotl protein sequences were also aligned to corresponding mouse proteins (Supplementary Figure S2A–C). Axolotl protein sequences were annotated, and relevant protein domains were identified manually and using the NCBI Conserved Protein Domain tool [21].

### 2.4. Antibody Selection

Strongly conserved epitopes in tau and BACE1 were predicted by aligning human protein sequences to the putative axolotl protein sequences. Commercial antibodies with immunogens containing epitopes with high similarity were obtained and tested in the axolotl. The tau antibody T46 (Thermo Fisher Scientific, Waltham, MA, USA; RRID: AB_2533025) recognizes amino acids 404–441 of the human MAPT protein—this epitope is 86% identical to the putative axolotl homolog. The BACE1 antibody 3C1C3 (Thermo Fisher Scientific, Waltham, MA, USA; RRID: AB_2608440) recognizes amino acids 112–324 of the human BACE1 protein—the immunogen sequence is 91% identical to the putative axolotl homolog. We ensured that the chosen antibodies also recognized the murine protein by evaluating prior publications using each antibody [22,23,24]. The antibodies were then used in immunoblotting and immunohistochemistry.

### 2.5. Preparation of Brain Tissue for Immunoblot Assay

To determine the approximate molecular weight of putative protein homologs in the axolotl, the brains of four adult axolotls (14–17 cm length; axolotl IDs 5–8) and one wild type 22-month-old C57BL/6J mouse were prepared as previously described [25]. Briefly, brains were homogenized in PBS with 1% each of P5726 and P0044 protease inhibitor cocktails (Sigma Aldrich, St. Louis, MO, USA). We utilized the soluble fraction for analysis [26]. Soluble protein concentrations were determined via BCA (bicinchoninic) assay (Fisher Scientific, Waltham, MA, USA), and 10 µg of protein lysate from each brain was adjusted to 1× with Laemmli buffer, boiled, loaded onto a 12% Tris-glycine gel (Invitrogen, Waltham, MA, USA), and subjected to SDS-PAGE (85 V for 30 min, immediately followed by 130 V for 80 min). Proteins were then transferred to nitrocellulose membranes (200 mA for 90 min).

### 2.6. Immunoblotting

The immunoblotting procedure by Xu et al. [27] was modified. Briefly, two identical sets of 4 axolotl and one B6 mouse brain were run in parallel on one gel and transferred to a nitrocellulose membrane. The membrane was then cut to create two panels—one to be probed with an antibody and the other to be probed with the target preabsorbed antibody. Membranes were blocked in 5% nonfat powdered milk (Adwin Scientific, Simi Valley, CA, USA) in TBS-T. For western blot pre-absorption, 1 µg of T46 (Thermo Fisher) was incubated with 20 µg of T43 4R Tau protein [28] (gift of Dr. Benoit Giasson), and 2 µg of 3C1C3 (Thermo Fisher) was incubated with 45 µg of recombinant human BACE1 (R&D Systems, Minneapolis, MN, USA) for one hour at room temperature with gentle agitation in 2 mL of 5% nonfat powdered milk in TBS-T alongside the same dilution of primary antibody without the target peptide. Each blot was then incubated overnight in either the primary antibody or the primary antibody preabsorbed with the target peptide (T43 or BACE1). Horseradish peroxidase-conjugated Donkey anti-mouse secondary antibody (Jackson ImmunoResearch, West Grove, PA, USA; RRID: AB_2340773) was used to visualize proteins through chemiluminescence using Western Lightning Plus-ECL (PerkinElmer, Shelton, CT, USA). Western Blot images were obtained using GeneTools by Syngene on a GeneSys imaging platform (Syngene, Frederick, MD, USA). After visualization, membranes were then cut horizontally at 40 kDa, and the portion containing the 15–40 kDa bands was re-blocked, re-probed with GAPDH (Meridian Life Science, Memphis, TN, USA; RRID: AB_2340773) and visualized as described above.

### 2.7. Immunohistochemistry

Four adult axolotl brains (14–17 cm; axolotl IDs 1–4) and four 5.5-month-old C57BL/6J mouse brains were collected and immersion-fixed in 10% neutral buffered formalin (Sigma Aldrich). Brains were bisected sagittally at the midline, processed as previously described [25], embedded in paraffin (Fisher), sectioned at 5 µm on the microtome, and mounted on Superfrost Plus precleaned microscope slides (Fisher). After drying overnight in a tissue oven, slides were deparaffinized and rinsed, and antigen retrieval was performed for 30 min in citrate buffer (10 mM Citrate, pH 6.0) in a pressure cooker (Bio SB, Santa Barbara, CA, USA). Slides were blocked in PBS-T with 2.5% normal horse serum (VectorLabs, Newark, CA, USA). Antibodies were initially tested at 1:50, 1:100, and 1:1000 to determine optimal primary antibody working concentrations. Experimental slides were then incubated overnight in the primary antibody (either T46, 1:100 or 3C1C3, 1:100) diluted in a blocking solution at room temperature. Slides were rinsed in PBS-T and blocked in 0.3% H_2_O_2_ for 30 min before being rinsed and incubated in a secondary antibody (VectorLabs ImmPRESS, horse anti-mouse). Slides were then rinsed in PBS-T, and a DAB (SeraCare Life Sciences, Milford, MA, USA) solution was applied to develop color. Slides were dehydrated in an ethanol gradient followed by xylene and mounted using Cytoseal 60 mounting media (Thermo Fisher). After drying for 48 h, slides were scanned on an Aperio AT2 slide scanner (Leica, Wetzlar, Germany). Images were processed using Aperio ImageScope (Leica Biosystems, Deer Park, IL, USA) and Photoshop (Adobe, San Jose, CA, USA) without adjusting color or contrast. Scale bars were added using ImageJ version 1.53t. Representative T46 and BACE1 images were selected. Technical replicates are available in Supplementary Data (Supplementary Figures S3 and S4). To ensure the secondary antibody did not cross-react with endogenous axolotl or mouse proteins, a secondary-only control slide was run without the primary antibody alongside slides stained with primary antibodies T46 and 3C1C3. To visualize the secondary-only slide, all slides in this experiment were counterstained with hematoxylin (Sigma) (Supplementary Figure S1). For this experiment, all axolotl tissues were stained at one time, and all mouse tissues were stained at one time, with the secondary-only control being the last slide washed in each batch.

## 3. Results

### A Tau-like Protein Is Expressed in the Axolotl Brain

Human tau, encoded by the *MAPT* gene, is a protein that normally binds tubulin and facilitates microtubule stabilization; however, it can pathologically aggregate inside neurons and form neurofibrillary tangles in Alzheimer’s disease (AD) and many cases of frontotemporal dementia (FTD) [29]. Tau is widely conserved across a variety of species, likely due to its critical role in stabilizing microtubules [30]; therefore, we predicted high levels of homology between humans and axolotls.

**Figure 1:**
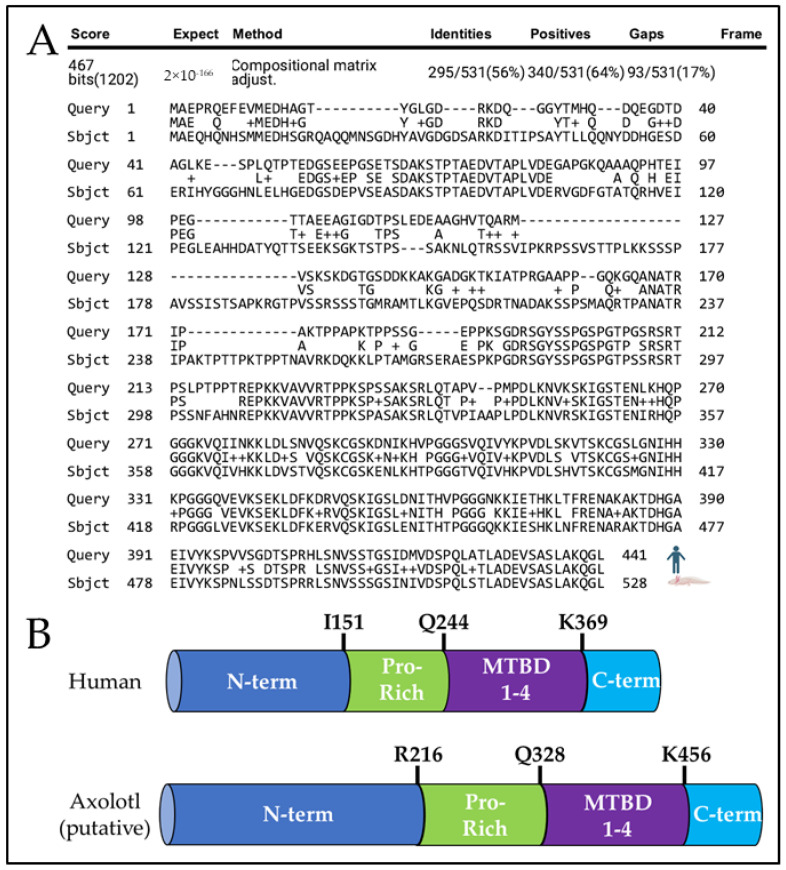
Comparison of human tau and putative axolotl tau. (**A**) Protein alignment of 2N4R human tau (top) and putative axolotl tau (bottom). (**B**) Identification of axolotl tau protein domains. Axolotl tau contains a putative N-terminus region (N-term, Dark Blue), a putative Proline-rich domain (Pro-Rich, Green), a microtubule-binding domain (MTBD 1–4, Purple), and a putative C-terminal domain (C-term, Light Blue).

Using the Axolotl-Omics.org tBlastN function [18] and the NCBI’s protein–protein BLAST alignment tool [20], we identified an axolotl sequence homologous to human *MAPT* and aligned its putative protein sequence to human tau protein (Figure 1A), and protein sequence alignment to murine tau (Supplementary Figure S2A). We predict axolotl tau is 528 amino axolotl acids long, which is only 56% identical to human tau when comparing the entire protein. While human tau contains an N-terminus region extending from M1 to I151, a Proline-rich domain from I151 to Q244, a microtubule-binding domain from Q244 to N368, and a C-terminus region from K369 to L441 [31], the putative axolotl tau contains an N-terminus region extending from M1 to D215, a Proline-rich domain from R216 to R327, a microtubule-binding domain from Q328 to Q455, and a C-terminus from K456 to L528 (Figure 1B). Axolotl tau is ~23% comprised of phosphorylation-prone amino acids serine (S), threonine (T), and tyrosine (Y) compared to ~19% in human 2N4R Tau (Reviewed in [32]). In humans, the proline-rich and microtubule-binding domains are implicated in tau protein function and aggregation [33]. The putative axolotl proline-rich region is composed of ~16% proline compared to the average of ~5% proline composition of all axolotl proteins [17] and ~24% in the proline-rich region of human 2N4R MAPT [34]. The putative microtubule-binding domain of axolotl tau appears highly homologous, with ~81% sequence identity to human tau and four tubulin-binding motifs, likely reflecting conservation of tau function across species.

**Figure 2:**
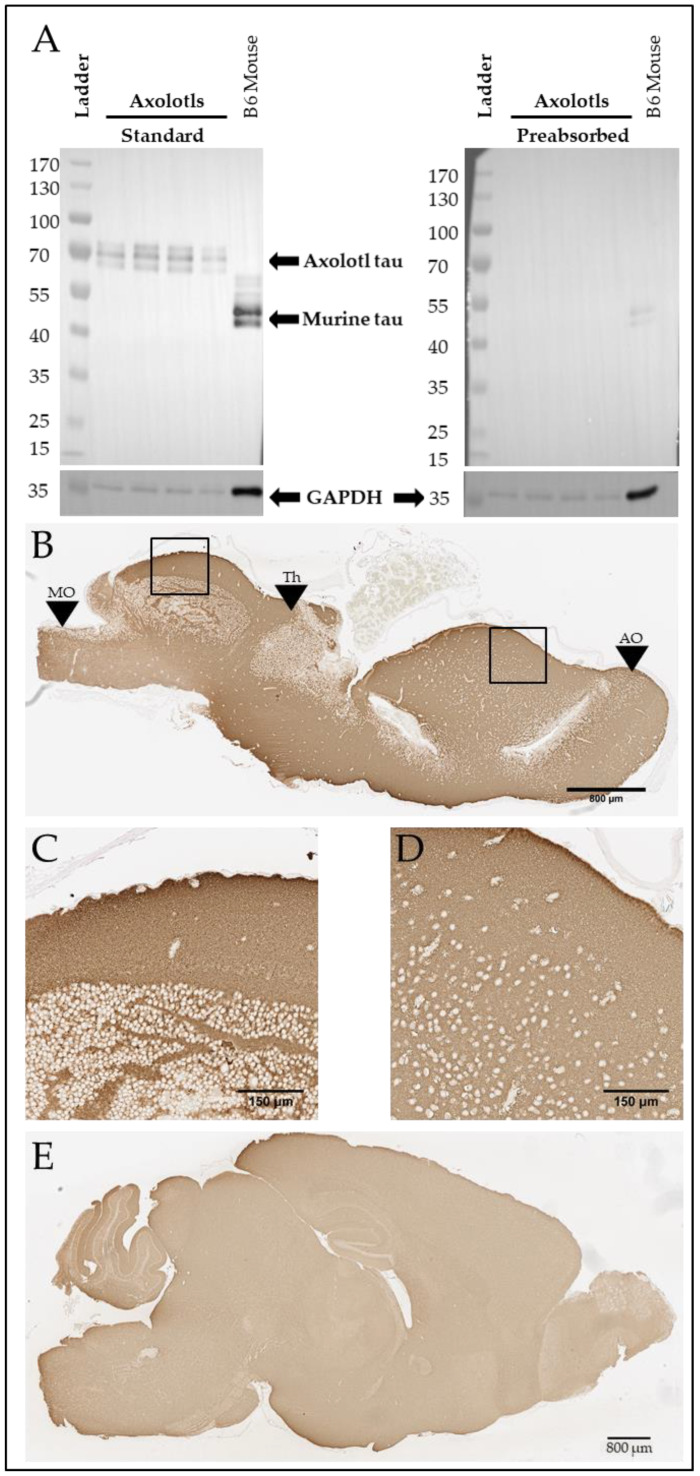
Axolotls express a putative tau protein in the brain. (**A**) Western blot demonstrating T46-positive signal at 45–60 kDA (mouse) and 65–75 kDa (axolotl) and GAPDH-positive signal at 36 kDa in both axolotl and mouse brain. T46 target preabsorption demonstrates antibody specificity. Both images were obtained from the same exposure. (**B**) Representative T46 immunostaining in sagittal sections of axolotl brain. Scale bar: 800 µm. Black boxes denote locations of (**C**,**D**). Black arrows: MO denotes the medulla oblongata; Th denotes the thalamus; AO denotes the accessory olfactory bulb. The accessory olfactory bulb is most anterior, and the medulla oblongata is most posterior. (**C**) 20× image of T46-positive immunostaining in the axolotl dorsal rhombencephalon. Scale bar: 150 µm. (**D**) 20× image of T46-positive immunostaining in the axolotl dorsal pallium. Scale bar: 150 µm. (**E**) Representative T46 immunostaining in sagittal section of B6 mouse brain. Scale bar: 800 µm.

To further explore tau homology across species, we ran a western blot on the soluble fraction of four axolotl brains and one mouse brain using anti-tau T46 (Thermo Fisher), a monoclonal antibody targeting the highly conserved tau C-Terminus. While mouse tau appears at approximately 45–60 kDa, as previously reported [22], axolotl tau appears to migrate at 65–75 kDa (Figure 2A, left). To ensure that bands recognized using the antibody in the axolotl brain were specific, we preabsorbed the antibody with T43 0N4R tau [28] (gift of Dr. Benoit Giasson), which strikingly reduced the signal (Figure 2A, right). Given the lack of background on the standard T46 western blot and the loss of signal with preabsorption (Figure 2A), we conclude that the signal observed on the western blot is likely an axolotl tau homolog. We then immunostained four wild-type adult axolotl (Figure 2B) and three C57BL/6J mouse brains (Figure 2C) with T46. As expected, diffuse Tau46 immunostaining was found throughout the axolotl brain, which was similarly observed throughout the murine brain tissue.

**Figure 3:**
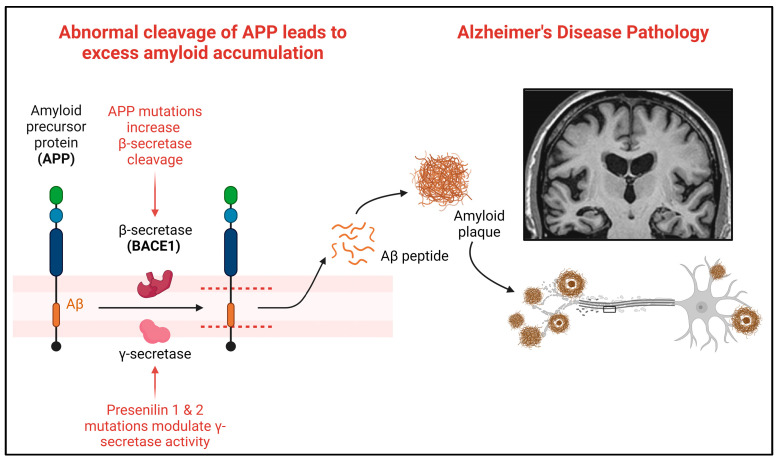
Amyloid precursor protein and the cleavage sites for amyloid in AD. Abnormal cleavage of APP leads to excess amyloid-β accumulation into amyloid plaques. Mutations in APP increase the β-secretase cleavage of APP into amyloid-β, driving the pathogenic accumulation of amyloid into plaques. Created with Biorender.com accessed on 5 February 2024.

In humans, amyloid precursor protein (APP) is encoded using the *APP* gene and cleaved into amyloid-β (Aβ) peptides, including Aβ42 [6]. Aβ42 accumulates and forms amyloid plaques, a hallmark neuropathological feature of AD [22]. Over 30 mutations in APP have been described as pathogenic in AD (https://www.alzforum.org/mutations/app, accessed on 23 January 2024). In humans, APP is sequentially cleaved, first via β-secretase (encoded by the *BACE1* gene) and then through γ-secretase to produce Aβ peptides [35] (Figure 3).

**Figure 4:**
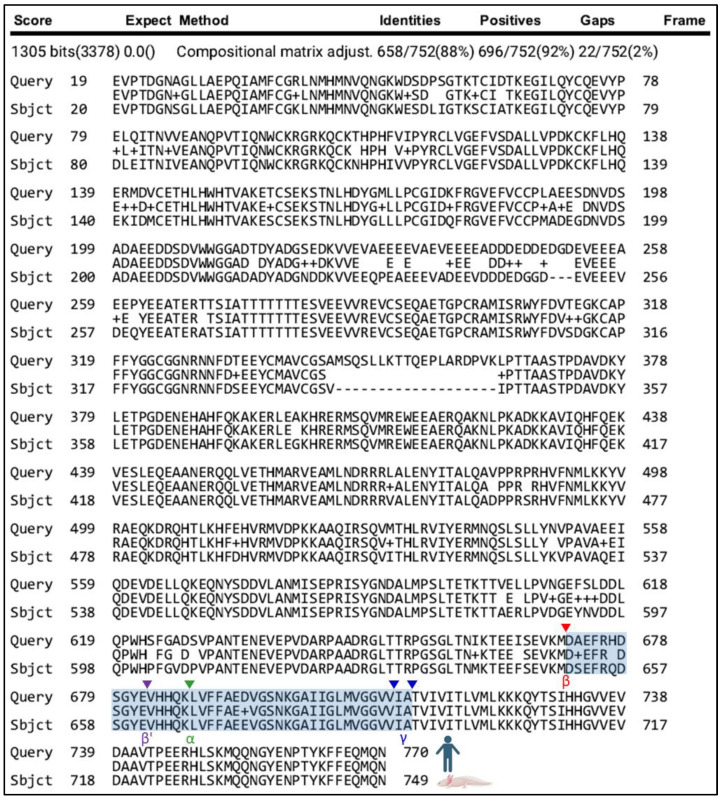
Amyloid precursor protein alignment (human on top, axolotl on bottom). The region of APP that encodes Aβ is highlighted in blue [38]. β-secretase cleavage sites β (Red), β′ (Purple), α-secretase cleavage site α (Green), and γ-secretase cleavage site γ (Blue) are denoted using the arrowheads. Two γ-secretase cleavage sites are shown because γ-secretase can cleave both Aβ-40 and Aβ-42.

Since APP processing is widely accepted as a critical factor in the development of AD [2,36], we sought to determine if critical players in this process were conserved between axolotl and humans. We identified a putative axolotl APP protein and aligned the sequence to human APP (Figure 4) and mouse APP (Supplementary Figure S2B) using a compositional matrix-adjusted sequence alignment [20]. We then further compared the region of human APP that gives rise to amyloid-β peptides for homology with the putative axolotl protein sequence. Major β-secretase cleavage sites (β and β′), α-secretase, and γ-secretase cleavage sites (α and γ) in human APP appear homologous to the axolotl sequence—the putative Aβ-encoding region is 93% identical between humans and axolotl (Figure 4)). The sequence between 1–17 in human APP encodes a signal peptide [37]; however, the putative axolotl sequence lacked significant homology within this sequence. Additionally, the OX-2 domain typically found in the longest version of human APP (amino acids 341–366) was largely absent (amino acids 339–344) from the putative axolotl APP protein [37].

**Figure 5:**
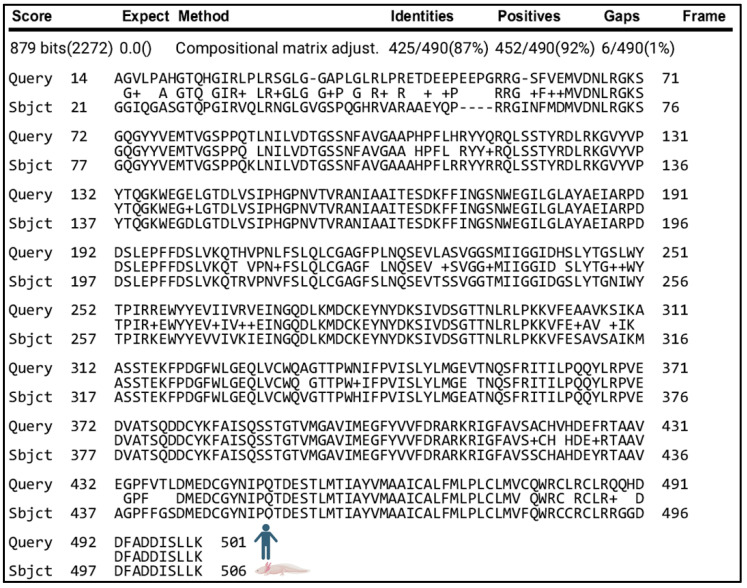
Protein alignment of human BACE1 (top) and putative axolotl BACE1 (bottom) shows that they share 87% of amino acids between human BACE1 amino acids 14–501 and the putative axolotl BACE1 amino acids 21–506.

Given the similarity of cleavage sites between the putative axolotl APP protein and its human counterpart, we predicted that axolotls contained secretases responsible for cleaving APP into amyloid-β peptides, as observed in humans. We identified a putative axolotl BACE1 protein and aligned the putative protein sequence to human BACE1 (Figure 5) and mouse BACE1 (Supplementary Figure S2C). *BACE1* encodes the enzyme β-secretase 1, which cleaves human APP at the β and β′ sites (Figure 4) and is highly similar between humans and axolotls. We found that 87% of the amino acids were identical between human BACE1 amino acids 14–501 and the putative axolotl BACE1 amino acids 21–506. (Figure 5). There is extensive conservation between the putative axolotl BACE1 and the catalytic, transmembrane, and cytosolic domains (amino acids 47–501 in human BACE1 [39]), including the aspartyl active sites (human D93, D289 vs. axolotl D98, D294).

**Figure 6:**
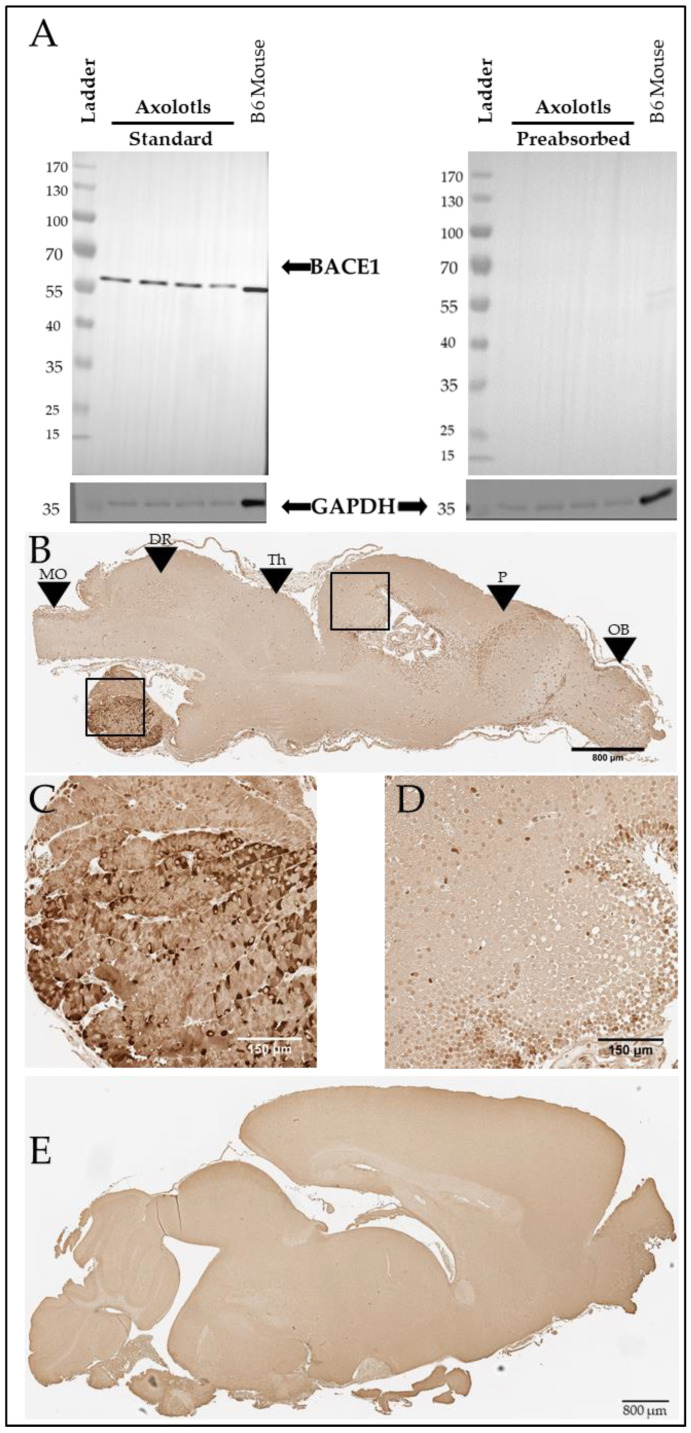
Axolotls express a putative BACE1 protein in the brain. (**A**) Western blot showing BACE1 signal at approximately 60 kDa and a GAPDH signal at 36 kDa in both axolotl and mouse brains. BACE1 target preabsorption control demonstrates antibody specificity in axolotl and mouse brains. (**B**) Representative BACE1 immunostaining in sagittal section of axolotl brain. Scale bar: 800 µm. Black boxes denote locations of (**C**,**D**). Black arrows: MO denotes medulla oblongata, DR denotes dorsal rhombencephalon, Th denotes the thalamus, P denotes the pallium, and OB denotes the olfactory bulb. The olfactory bulb is most anterior, and the medulla oblongata is most posterior. (**C**) 20× image of BACE1-positive immunostaining in the axolotl pituitary. Scale bar: 150 µm. (**D**) 20× image of BACE1-positive immunostaining in the axolotl dorsal pallium. Scale bar: 150 µm. (**E**) BACE1 immunostaining in sagittal section of B6 mouse brain. Scale bar: 800 µm.

We further investigated the putative axolotl BACE1 using western blotting. Using antibody 3C1C3, we demonstrate that axolotls express a putative BACE1 protein at approximately 60 kDa (Figure 6A, Left). The axolotl BACE1 sequence we identified has a predicted molecular weight of 56 kDa [40]. In mammals, BACE1 undergoes glycosylation and other post-translational modifications [41], which may contribute to the putative axolotl BACE1 running slightly higher than predicted based on sequence alone. The putative axolotl BACE1 band migrated at approximately the same size as the murine BACE1 band, and minimal background staining was observed on the blot itself, supporting the likelihood that the detected band in axolotl brain lysate is indeed BACE1 (Figure 6A). For further confirmation, we performed pre-absorption of the antibody with human recombinant BACE1, which eliminated the observed signal (Figure 6A), suggesting that this band is specific. We further analyzed BACE1 in the axolotl via immunohistochemistry using 3C1C3 (Thermo Fisher) and demonstrated immunopositive staining in axolotl brain sections (Figure 6B–D), revealing a similar staining pattern to that observed in murine brain (Figure 6E).

## 4. Discussion

While neurodegenerative diseases have never been reported in the axolotl, our analysis of putative axolotl proteins homologous to proteins implicated in human neurodegenerative diseases suggests that the axolotl may have the baseline capacity to develop neurodegenerative diseases similar to those that occur in humans. Our long-term goal is to identify neuroresilience and neurorestorative pathways that may be therapeutically exploited to combat neurodegeneration in Alzheimer’s Disease and related tauopathies. In the current report, we sought to determine if axolotls possess the genetic capacity to potentially serve as a model for neuroregeneration in the context of neurodegenerative proteinopathies. We began our study with three critical proteins involved in Alzheimer’s Disease—tau, APP, and BACE1. Based on our analysis of the published axolotl genome, axolotls possess homologs for *MAPT*, *APP*, and *BACE1*. Profound protein sequence similarity and immunopositive staining using currently available antibodies targeting homologous protein sequences supports the notion that these axolotl proteins are expressed and may serve similar functions as their human counterparts.

The putative axolotl tau protein identified here and the human 2N4R tau protein are highly homologous within the microtubule-binding domains and C-terminus but show a lower level of homology in the putative N-terminus and proline-rich domain. These differences help account for the larger size of the putative axolotl tau protein. The larger putative axolotl tau is supported by the difference in T46 staining in Figure 2A, where the axolotl T46-positive bands migrated at 65–75 kDa compared to 45–60 kDa in the mouse. Interestingly, the axolotl T46 bands in Figure 2A appear heavier than the predicted 56 kDa molecular weight of the putative axolotl tau amino acid sequence we calculated using The Sequence Manipulation Suite [40]. This may be due to phosphorylation or other post-translational modifications, which can affect protein mobility in western blots. In humans, the *MAPT* transcript is alternatively spliced, generating six different major isoforms in the brain that vary in number of N-terminal regions and microtubule binding repeats; however, our analysis focused on the longest isoform, 2N4R tau, which is 441 amino acids long (reviewed in [42]). While multiple bands are present, Tau46 recognizes all six major isoforms of human tau found in the brain, regardless of phosphorylation, so it is possible that the different bands in the axolotl are simply different isoforms. The T46 signal replicates previously published uses of this antibody in mice [22,23]. Interestingly, despite the proline-rich region and microtubule-binding region being central to the normal microtubule binding function and abnormal aggregation of tau (reviewed in [42]), we identify a lower proportion of proline residues within the putative axolotl proline-rich region of tau and human tau proline-rich region, at ~16% versus ~24%. In contrast, the putative axolotl tau sequence we identified also has a higher level of the phosphorylation-prone amino acids serine (S), threonine (T), and tyrosine (Y), at ~23% compared with ~19% in human 2N4R tau (Reviewed in [32]). However, exploration of axolotl *Mapt* splicing and protein post-translational modifications fall outside the scope of the current report.

Amyloid precursor protein (APP) and the enzymes that it interacts with are well studied in humans. Strong genetic homology and immunopositive staining of BACE1 using currently available antibodies indicate that APP cleavage biology may be similar between axolotl and humans. In-depth characterization studies of APP biology in the axolotl may be within the reach of current scientific tools.

We show that currently available antibodies used in human or mouse studies recognize epitopes highly conserved in the axolotl, providing the tools to interrogate the expression and localization of key neurodegeneration-associated proteins without synthesizing novel antibodies. Furthermore, tools that induce proteinopathies in mice, such as those used in seeding and spreading studies [43], may be useful in the axolotl as a method of inducing tauopathy and amyloidosis. Given the high degree of similarity between the putative axolotl tau microtubule-binding domain and studies that have previously shown that truncated tau proteins composed of the human microtubule-binding domain are sufficient to induce the formation of neurofibrillary tangles in vivo, inducing tauopathy in the axolotl may be within reach [44,45]. One caveat is that tau, APP, and secretases do not function in a vacuum, and it is possible that other known or unknown proteins may differentially interact with these proteinopathy-related proteins in axolotls versus humans. Going forward, work should seek to characterize other neurodegeneration-associated homologs in the axolotl to position the field to determine if axolotls may have the capacity to be manipulated to study additional pathways associated with a large number of human neurodegenerative diseases in the context of the axolotl’s unique regenerative ability. If axolotl homologs of fibrilization-prone proteins can be induced to fibrillize in vitro or in vivo in the axolotl, examination of the response of axolotl glia and inflammasome may help explain axolotl neuroresilience.

While one may argue that an aquatic salamander is not relevant for the study of human diseases, many other organisms can function as natural or induced models of proteinopathies and have demonstrated utility in understanding the development of these disorders [46]. Many models of neurodegenerative diseases exist in other vertebrates, but none possess the same regenerative abilities or potential utility of the axolotl in understanding molecular mechanisms of neuroresilience. Adding the axolotl to the collection of models currently used to study neurodegeneration would be unique, as it would be the only genetic model organism with such documented, advanced neuroregenerative capabilities. Analyzing the axolotl’s physiological responses to an attempt to induce proteinopathies would help answer key questions about the extent of axolotl neuroprotection to disease and whether the axolotl is fundamentally protected against the development of neuropathology, can delay or combat the development of, or can regenerate cells lost to neurodegeneration. The current report lays critical groundwork for the field to undertake these studies and suggests that axolotls could prove useful in searching for therapeutic avenues to regenerate cells lost to neurodegenerative disease.

## Figures and Tables

**Table 1:** Summary of Alignment Results.

Human Gene	Protein	Score	E-Value	Percent Identical	Percent Functionally Equivalent	Gaps
*MAPT*	Microtubule-associated protein tau	467	2 × 10^−166^	56% (295/531)	64% (340/531)	17% (93/531)
*APP*	Amyloid precursor protein	1305	0.0	88% (658/752)	92% (696/752)	2% (22/752)
*BACE1*	β-secretase 1	879	0.0	87% (425/490)	92% (452/490)	1% (6/490)

## Data Availability

All data necessary to interpret this work are contained within the manuscript and Supplementary Materials.

## Supplementary Materials

The following supporting information can be downloaded at https://www.mdpi.com/article/10.3390/genes15030310/s1, Figure S1: Secondary-only IHC control, Figure S2A: Alignment of Mouse MAPT and Putative Axolotl MAPT, Figure S2B: Alignment of Mouse APP and Putative Axolotl APP, Figure S2C: Alignment of Mouse BACE1 and Putative Axolotl BACE1, Figure S3: T46 Immunohistochemistry Technical Replicates, Figure S4: BACE1 Immunohistochemistry Technical Replicates; Table S1: Reference Sequences for Genes of Interest, Table S2: Accession Numbers for Identified Axolotl cDNA and Putative Protein Sequences.
