# Supplementary material for: Identification and Analysis of Axolotl Homologs for Proteins Implicated in Human Neurodegenerative Proteinopathies
Source: Genes (Basel). 2024 Feb 28;15(3):310. doi: 10.3390/genes15030310 (PMC10969905; doi:10.3390/genes15030310)
Supplement: Supplementary file 1 [file genes-15-00310-s001.zip › genes-2884556-supplementary.pdf]

Supplementary Figures: Identification and Analysis of Axolotl Homologs for Proteins Implicated in Human Neurodegenerative Proteinopathies

Supplemental Table S1. Reference Sequences for Genes of Interest.

Gene	Protein	Associated Diseases	Human Reference Sequence	Mouse Reference Sequence
MAPT	Microtubule associated protein tau	AD, FTD	NP_005901.2	P10637-2
APP	Amyloid precursor protein	AD	NP_000475.1	P12023
BACE1	Beta-secretase 1	AD	NP_036236.1	P56818

Supplemental Table S2. Accession numbers for identified axolotl cDNA and putative protein sequences

Gene Name	Protein Name	Axolotl-Omics.org Reference Sequence
Mapt	MAPT	AMEXTC_0340000005280
App	APP	AMEXTC_0340000235000
Bace1	BACE1	AC_02200039355.1

1. **Supplementary Figure S1:** Secondary-Only IHC Control

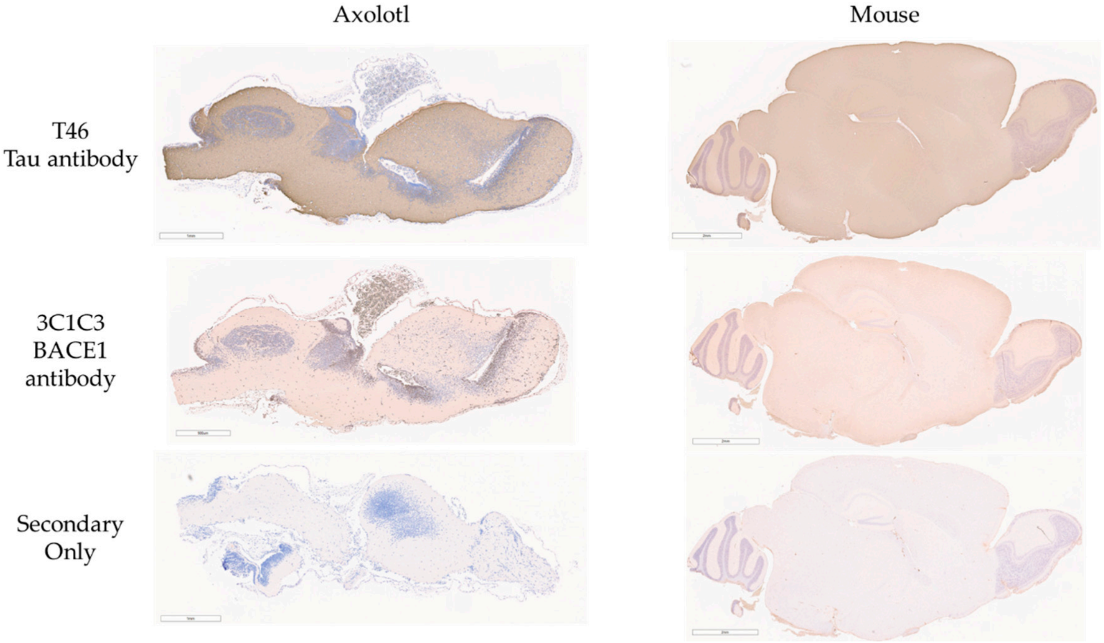

ImmPRESS Horse anti-Mouse Kit only IHC control. The absence of DAB signal in the secondary-only tissues demonstrates that our secondary antibody kit is specific to our primary antibody and does not interact with endogenous proteins in axolotl brain tissue.

Axolotl and Mouse Protein Alignments

a. **Supplementary Figure S2A.** Alignment of Mouse MAPT (Top) and putative Axolotl MAPT (bottom).

```
Score           Expect   Method                         Identities     Positives      Gaps
461 bits(1185)  4e-164   Compositional matrix adjust.   289/533(54%)   337/533(63%)   108/533(20%)

Query  1    MADPRQEFDTMEDHAGD----------------------------YTLLQ---DQEGDMD  29
            MA+  Q    MEDH+G                             YTLLQ   D  G+ D
Sbjct  1    MAEQHQNHSMMEDHSGRQAQQMNSGDHYAVGDGDSARKDITIPSAYTLLQQNYDDHGESD  60

Query  30   --------HGLKESPPQPPADDGAEEPGSETSDAKSTPTAEDVTAPLVDERAPDKQAAAQ  81
                    H L     +   +DG++EP SE SDAKSTPTAEDVTAPLVDER  D   A Q
Sbjct  61   ERIHYGGGHNL-----ELHGEDGSDEPVSEASDAKSTPTAEDVTAPLVDERVGDFGTATQ  115

Query  82   PHTEIPEGITA-----------EEAGIGDTPN--QEDQAAGHVTQAR-------------  115
             H EIPEG+ A           E++G   TP+  +  Q    V   R
Sbjct  116  RHVEIPEGLEAHHDATYQTTSEEKSGKTSTPSSAKNLQTRSSVIPKRPSSVSTTPLKKSS  175

Query  116  -----------------VASK-DRTGNDEKKAKGADGKTGAKIATPRGAASPAQKGTSNA  157
                             V+S+   TG      KG + ++    A  + + S AQ+  +NA
Sbjct  176  SPAVSSISTSAPKRGTPVSSRSSSTGMRAMTLKGVEPQSDRTNADAKSSPSMAQRTPANA  235

Query  158  TRIPA------------------KTTPSPKTPPGSGEPPKSGERSGYSSPGSPGTPGSRS  199
            TRIPA                  K  P+        E PK G+RSGYSSPGSPGTP SRS
Sbjct  236  TRIPAKTPTTPKTPPTNAVRKDQKKLPTAMGRSERAESPKPGDRSGYSSPGSPGTPSSRS  295

Query  200  RTPSLPTPPTREPKKVAVVRTPPKSPSASKSRLQTAPV--PMPDLKNVRSKIGSTENLKH  257
            RTPS      REPKKVAVVRTPPKSP+++KSRLQT P+  P+PDLKNVRSKIGSTEN++H
Sbjct  296  RTPSSNFAHNREPKKVAVVRTPPKSPASAKSRLQTVPIAAPLPDLKNVRSKIGSTENIRH  355

Query  258  QPGGGKVQIINKKLDLSNVQSKCGSKDNIKHVPGGGSVQIVYKPVDLSKVTSKCGSLGNI  317
            QPGGGKVQI++KKLD+S VQSKCGSK+N+KH PGGG+VQIV+KPVDLS VTSKCGS+GNI
Sbjct  356  QPGGGKVQIVHKKLDVSTVQSKCGSKENLKHTPGGGTVQIVHKPVDLSHVTSKCGSMGNI  415

Query  318  HHKPGGGQVEVKSEKLDFKDRVQSKIGSLDNITHVPGGGNKKIETHKLTFRENAKAKTDH  377
            HH+PGGG VEVKSEKLDFK+RVQSKIGSL+NITH PGGG KKIE+HKL FRENA+AKTDH
Sbjct  416  HHRPGGGLVEVKSEKLDFKERVQSKIGSLENITHTPGGGQKKIESHKLNFRENARAKTDH  475

Query  378  GAEIVYKSPVVSGDTSPRHLSNVSSTGSIDMVDSPQLATLADEVSASLAKQGL  430
            GAEIVYKSP +S DTSPR LSNVSS+GSI++VDSPQL+TLADEVSASLAKQGL
Sbjct  476  GAEIVYKSPNLSSDTSPRRLSNVSSSGSINIVDSPQLSTLADEVSASLAKQGL  528
```

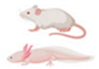

b. **Supplementary Figure S2B.** Alignment of Mouse APP (top) and putative Axolotl APP (bottom).

```
Score            Expect  Method                        Identities      Positives       Gaps
1296 bits(3354)  0.0     Compositional matrix adjust.  656/752(87%)    693/752(92%)    22/752(2%)

Query  19    EVPTDGNAGLLAEPQIAMFCGKLNMHMNVQNGKWESDPSGTKTCIGTKEGILQYCQEVYP  78
             EVPTDGN+GLLAEPQIAMFCGKLNMHMNVQNGKWESD  GTK+CI TKEGILQYCQEVYP
Sbjct  20    EVPTDGNSGLLAEPQIAMFCGKLNMHMNVQNGKWESDLIGTKSCIATKEGILQYCQEVYP  79

Query  79    ELQITNVVEANQPVTIQNWCKRGRKQCKTHTHIVIPYRCLVGEFVSDALLVPDKCKFLHQ  138
             +L+ITN+VEANQPVTIQNWCKRGRKQCK H HIV+PYRCLVGEFVSDALLVPDKCKFLHQ
Sbjct  80    DLEITNIVEANQPVTIQNWCKRGRKQCKNHPHIVVPYRCLVGEFVSDALLVPDKCKFLHQ  139

Query  139   ERMDVCETHLHWHTVAKETCSEKSTNLHDYGMLLPCGIDKFRGVEFVCCPLAEESDSVDS  198
             E++D+CETHLHWHTVAKE+CSEKSTNLHDYG+LLPCGID+FRGVEFVCCP+A+E D+VDS
Sbjct  140   EKIDMCETHLHWHTVAKESCSEKSTNLHDYGLLLPCGIDQFRGVEFVCCPMADEGDNVDS  199

Query  199   ADAEEDDSDVWWGGADTDYADGGEDKVVEVAEEEEVADVEEEEADDDEDVEDGDEVEEEA  258
             ADAEEDDSDVWWGGAD DYADG +DKVVE   E E    +EE  DD++  +   EVEEE
Sbjct  200   ADAEEDDSDVWWGGADADYADGNDDKVVEEQPEAEEEVADEEVDDDEDGGD---EVEEEV  256

Query  259   EEPYEEATERTTSTATTTTTTTESVEEVVREVCSEQAETGPCRAMISRWYFDVTEGKCVP  318
             +E YEEATER TS ATTTTTTTESVEEVVREVCSEQAETGPCRAMISRWYFDV++GKC P
Sbjct  257   DEQYEEATERATSIATTTTTTTESVEEVVREVCSEQAETGPCRAMISRWYFDVSDGKCAP  316

Query  319   FFYGGCGGNRNNFDTEEYCMAVCGSVSTQSLLKTTSEPLPQDPDKLPTTAASTPDAVDKY  378
             FFYGGCGGNRNNFD+EEYCMAVCGSV                   +PTTAASTPDAVDKY
Sbjct  317   FFYGGCGGNRNNFDSEEYCMAVCGSV-------------------IPTTAASTPDAVDKY  357

Query  379   LETPGDENEHAHFQKAKERLEAKHRERMSQVMREWEEAERQAKNLPKADKKAVIQHFQEK  438
             LETPGDENEHAHFQKAKERLE KHRERMSQVMREWEEAERQAKNLPKADKKAVIQHFQEK
Sbjct  358   LETPGDENEHAHFQKAKERLEGKHRERMSQVMREWEEAERQAKNLPKADKKAVIQHFQEK  417

Query  439   VESLEQEAANERQQLVETHMARVEAMLNDRRRLALENYITALQAVPPRPHHVFNMLKKYV  498
             VESLEQEAANERQQLVETHMARVEAMLNDRRR+ALENYITALQA PPR  HVFNMLKKYV
Sbjct  418   VESLEQEAANERQQLVETHMARVEAMLNDRRRVALENYITALQADPPRSRHVFNMLKKYV  477

Query  499   RAEQKDRQHTLKHFEHVRMVDPKKAAQIRSQVMTHLRVIYERMNQSLSLLYNVPAVAEEI  558
             RAEQKDRQHTLKHF+HVRMVDPKKAAQIRSQV+THLRVIYERMNQSLSLLY VPAVA+EI
Sbjct  478   RAEQKDRQHTLKHFDHVRMVDPKKAAQIRSQVITHLRVIYERMNQSLSLLYKVPAVAQEI  537

Query  559   QDEVDELLQKEQNYSDDVLANMISEPRISYGNDALMPSLTETKTTVELLPVNGEFSLDDL  618
             QDEVDELLQKEQNYSDDVLANMISEPRISYGNDALMPSLTETKTT E LPV+GE+++DDL
Sbjct  538   QDEVDELLQKEQNYSDDVLANMISEPRISYGNDALMPSLTETKTTAERLPVDGEYNVDDL  597

Query  619   QPWHPFGVDSVPANTENEVEPVDARPAADRGLTTRPGSGLTNIKTEEISEVKMDAEFGHD  678
             QPWHPFGVD VPANTENEVEPVDARPAADRGLTTRPGSGLTN+KTEE SEVKMD+EF  D
Sbjct  598   QPWHPFGVDPVPANTENEVEPVDARPAADRGLTTRPGSGLTNMKTEEFSEVKMDSEFRQD  657

Query  679   SGFEVRHQKLVFFAEDVGSNKGAIIGLMVGGVVIATVIVITLVMLKKKQYTSIHHGVVEV  738
             SG+EV HQKLVFFAE+VGSNKGAIIGLMVGGVVIATVIVITLVMLKKKQYTSIHHGVVEV
Sbjct  658   SGYEVHHQKLVFFAEEVGSNKGAIIGLMVGGVVIATVIVITLVMLKKKQYTSIHHGVVEV  717

Query  739   DAAVTPEERHLSKMQQNGYENPTYKFFEQMQN  770
             DAAVTPEERHLSKMQQNGYENPTYKFFEQMQN
Sbjct  718   DAAVTPEERHLSKMQQNGYENPTYKFFEQMQN  749
```

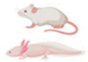

c. **Supplementary Figure S2C.** Alignment of Mouse BACE1 (top) and putative Axolotl BACE1 (bottom).

```
Score            Expect  Method                         Identities       Positives        Gaps
870 bits(2247)   0.0     Compositional matrix adjust.   417/488(85%)     446/488(91%)     4/488(0%)

Query  15   GMLPAQGTHLGIRLPLRSGLA-GPPLGLRLPRETDEESEEPGRRGSFVEMVDNLRGKSGQ  73
            G+  A GT  GIR+ LR+GL  G P G R+ R  + +   P R  +F++MVDNLRGKSGQ
Sbjct  22   GIQGASGTQPGIRVQLRNGLGVGSPQGHRVARAAEYQ---PRRGINFMDMVDNLRGKSGQ  78

Query  74   GYYVEMTVGSPPQTLNILVDTGSSNFAVGAAPHPFLHRYYQRQLSSTYRDLRKGVYVPYT  133
            GYYVEMTVGSPPQ LNILVDTGSSNFAVGAA HPFL RYY+RQLSSTYRDLRKGVYVPYT
Sbjct  79   GYYVEMTVGSPPQKLNILVDTGSSNFAVGAAAHPFLRRYYRRQLSSTYRDLRKGVYVPYT  138

Query  134  QGKWEGELGTDLVSIPHGPNVTVRANIAAITESDKFFINGSNWEGILGLAYAEIARPDDS  193
            QGKWEG+LGTDLVSIPHGPNVTVRANIAAITESDKFFINGSNWEGILGLAYAEIARPDDS
Sbjct  139  QGKWEGDLGTDLVSIPHGPNVTVRANIAAITESDKFFINGSNWEGILGLAYAEIARPDDS  198

Query  194  LEPFFDSLVKQTHIPNIFSLQLCGAGFPLNQTEALASVGGSMIIGGIDHSLYTGSLWYTP  253
            LEPFFDSLVKQT +PN+FSLQLCGAGF LNQ+E  +SVGG+MIIGGID SLYTG++WYTP
Sbjct  199  LEPFFDSLVKQTRVPNVFSLQLCGAGFSLNQSEVTSSVGGTMIIGGIDGSLYTGNIWYTP  258

Query  254  IRREWYYEVIIVRVEINGQDLKMDCKEYNYDKSIVDSGTTNLRLPKKVFEAAVKSIKAAS  313
            IR+EWYYEV+IV++EINGQDLKMDCKEYNYDKSIVDSGTTNLRLPKKVFE+AV +IK AS
Sbjct  259  IRKEWYYEVVIVKIEINGQDLKMDCKEYNYDKSIVDSGTTNLRLPKKVFESAVSAIKMAS  318

Query  314  STEKFPDGFWLGEQLVCWQAGTTPWNIFPVISLYLMGEVTNQSFRITILPQQYLRPVEDV  373
            STEKFPDGFWLGEQLVCWQ GTTPW+IFPVISLYLMGE TNQSFRITILPQQYLRPVEDV
Sbjct  319  STEKFPDGFWLGEQLVCWQVGTTPWHIFPVISLYLMGEATNQSFRITILPQQYLRPVEDV  378

Query  374  ATSQDDCYKFAVSQSSTGTVMGAVIMEGFYVVFDRARKRIGFAVSACHVHDEFRTAAVEG  433
            ATSQDDCYKFA+SQSSTGTVMGAVIMEGFYVVFDRARKRIGFAVS+CH HDE+RTAAV G
Sbjct  379  ATSQDDCYKFAISQSSTGTVMGAVIMEGFYVVFDRARKRIGFAVSSCHAHDEYRTAAVAG  438

Query  434  PFVTADMEDCGYNIPQTDESTLMTIAYVMAAICALFMLPLCLMVCQWRCLRCLRHQHDDF  493
            PF  +DMEDCGYNIPQTDESTLMTIAYVMAAICALFMLPLCLMV QWRC RCLR   DDF
Sbjct  439  PFFGSDMEDCGYNIPQTDESTLMTIAYVMAAICALFMLPLCLMVFQWRCCRCLRRGGDDF  498

Query  494  ADDISLLK  501
            ADDISLLK
Sbjct  499  ADDISLLK  506
```

2. Supplementary Figure S3. T46 Immunohistochemistry Technical Replicates
    a. T46 immunostaining in sagittal section of axolotl whole brain. Scale bars 800μm.

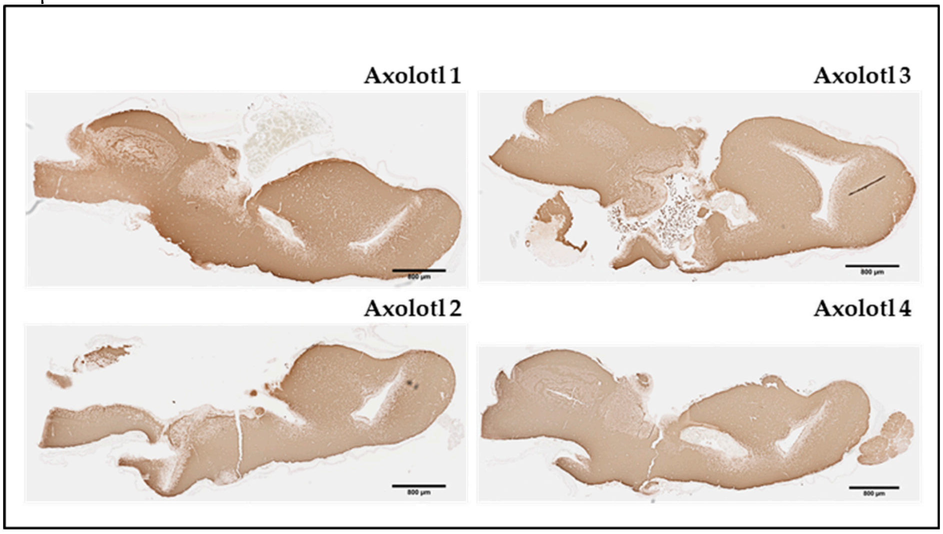

    b. T46 immunostaining in sagittal section of mouse whole brain. Scale bars 800μm.

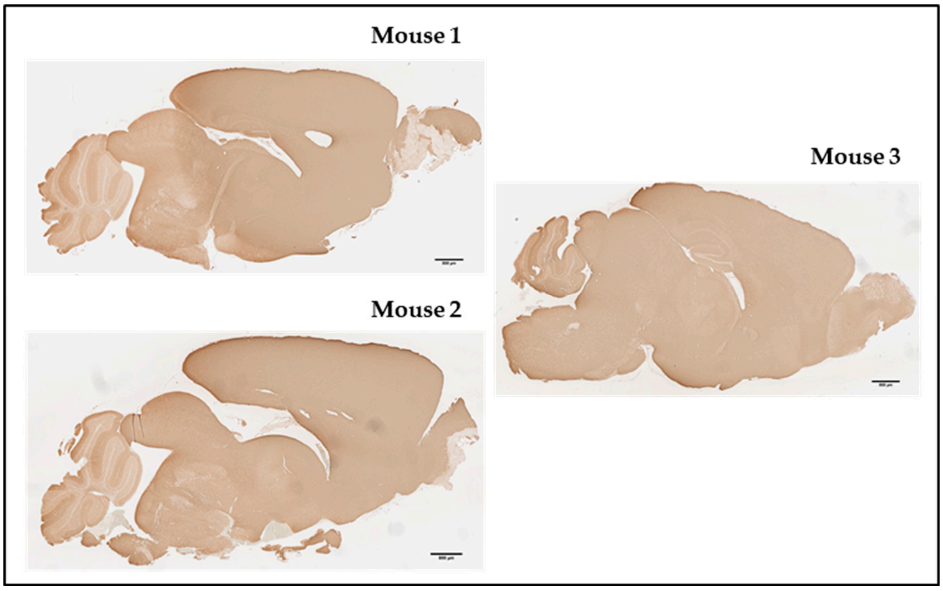

3. **Supplementary Figure S4:** BACE1 Immunohistochemistry Technical Replicates
    a. BACE1 immunostaining in sagittal section of axolotl whole brain. Scale bars 800µm.

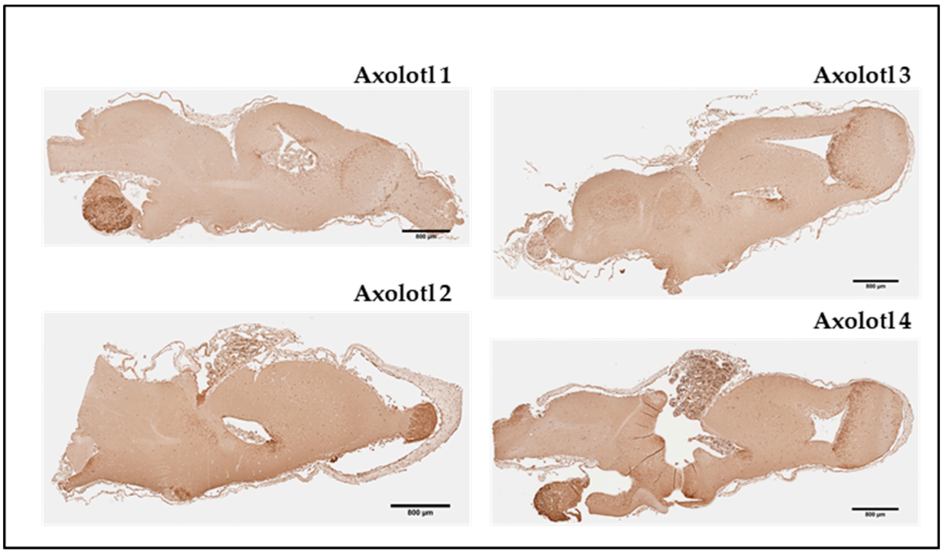

b. BACE1 immunostaining in sagittal section of mouse whole brain. Scale bars 800µm.

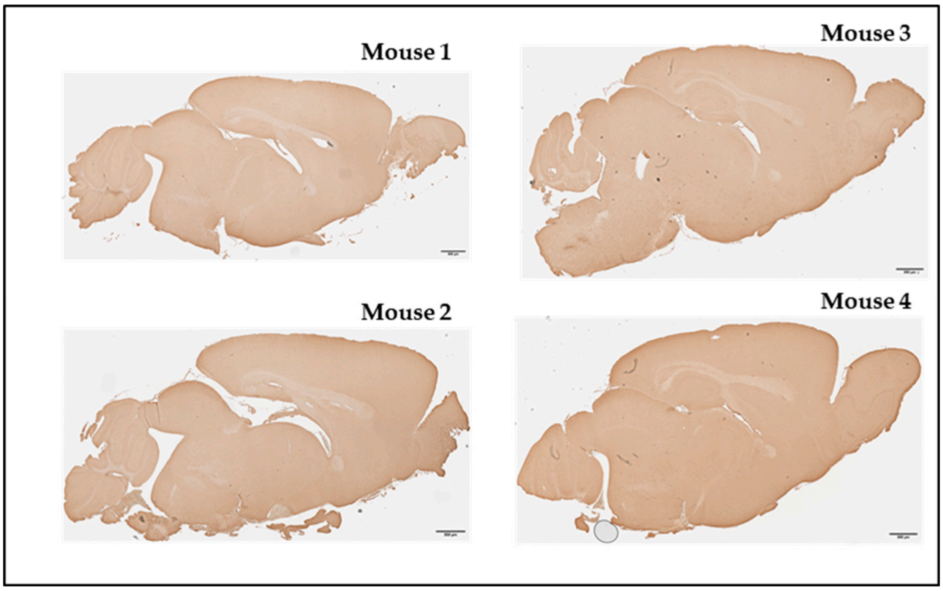
Mouse 1
Mouse 3
Mouse 2
Mouse 4
